# Mobile Exergames to Reduce Sedentary Time in Patients With Heart Failure: What Do Health Care Professionals Expect?

**DOI:** 10.2196/69126

**Published:** 2025-11-26

**Authors:** Leonie Klompstra, Anna Strömberg, Antoni Bayes-Genis, Maria Boldo, Beatriz González, Nuria Santaularia, Tiny Jaarsma

**Affiliations:** 1 Department of Health, Medicine and Caring Sciences, Linköping University, Linköping, 581 83, Sweden, 46 700896360; 2 Rehabilitation Service and Heart Failure Unit, Hospital Universitari Germans Trias i Pujol, Badalona, Spain; 3 Althaia, Xarxa Assistencial Universitària de Manresa, Manresa, Spain

**Keywords:** exergaming, heart failure, implementation, sedentary, serious games

## Abstract

Health care professionals emphasized that while exergaming holds promise as a motivational approach to supporting physical activity among patients with heart failure, its success depends on thoughtful integration into existing care pathways, personalization to patient needs, and addressing technological barriers.

## Introduction

Exergaming, which combines exercise and video gaming, has emerged as a promising intervention for decreasing older adults’ sedentary time [1]. Sedentary behavior is a significant concern in patients with heart failure (HF). Regular physical activity (PA) improves quality of life and physical function and reduces hospitalizations [2]. However, despite these benefits, many patients struggle to engage in sufficient PA due to various barriers, including physical limitations, lack of motivation, and psychological factors [2,3].

Exergaming can encourage PA among patients with cardiovascular disease [4]. In a randomized controlled trial (RCT) [5], we tested a commercial exergame platform on a television (Nintendo Wii). Participants expressed a desire to engage in exergaming outdoors and have the exergame tailored to their PA levels [6]. Based on these insights, we developed a mobile phone exergame [7,8] tailored to patients’ exercise capacity, and a new RCT [9] involving patients with HF is ongoing. Health care professionals’ (HCPs’) expectations regarding mobile exergaming are important, as they play a key role in recommending and supervising physical activities for patients with HF and for mobile exergame implementation in clinical practice. Given the lack of existing literature on HCPs’ expectations, this study’s purpose was to explore HCPs’ expectations regarding mobile exergaming as an approach to decreasing sedentary time in patients with HF.

## Methods

### Ethical Considerations

Ethical approval was not required for this study, as no sensitive or personally identifiable data were collected, in accordance with institutional guidelines. Verbal informed consent was obtained from all participants. Interview data were anonymized during transcription, no compensation was provided, and all procedures complied with institutional and GDPR (General Data Protection Regulation) guidelines [10-12].

### Study Design

A qualitative study with semistructured interviews was performed between September and October 2023 with 17 HCPs (9 cardiologists, 2 rehabilitation physicians, 5 nurses, and 1 physiotherapist) who were involved in the care of patients with HF at the cardiology departments of Althaia, Xarxa Assistencial Universitària de Manresa (Manresa, Spain) and Hospital Universitari Germans Trias i Pujol (Barcelona, Spain).

For semistructured interviews on HCPs’ experiences with sex differences in exercise among patients with HF, HCPs were asked one additional question to explore HCPs’ expectations regarding mobile exergaming as an approach to decreasing sedentary time in patients with HF (Multimedia Appendix 1). The interviews were conducted by a medical student (supervised by 2 experienced researchers [TJ and LK]), recorded, transcribed verbatim, and analyzed via thematic analysis [13].

## Results

**Figure 1.**
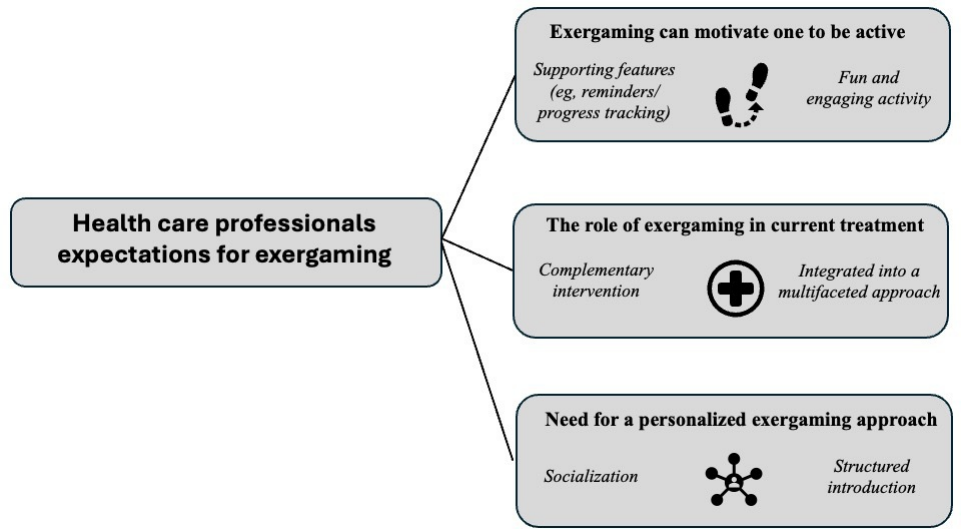
Themes (with strategies in italics) describing expectations that health care professionals had regarding exergaming as an approach to decreasing sedentary time in patients with heart failure.

Three key themes on HCPs’ expectations regarding mobile exergaming for patients with HF were found (Figure 1).

### Exergaming Can Motivate One to Be Active

One of the most expressed expectations was that exergaming may motivate patients with HF to engage in regular PA. The interactive and gamified nature of exergaming was seen as appealing, as it can transform PA from a chore into a fun and engaging activity. HCPs also highlighted mobile exergaming’s potential to support regular PA through reminders and progress tracking. They believed that such features could help patients maintain a consistent PA routine, which would ultimately lead to reduced sedentary time.

### Adding but Not Replacing

HCPs viewed exergaming as a complementary intervention rather than as a replacement for traditional PA advice or cardiac rehabilitation, acknowledging that while exergaming could offer benefits, it should not be seen as a stand-alone solution. Instead, it should be integrated into a broader, multifaceted approach to managing HF, potentially enhancing the effects of standard rehabilitation.

### Need for a Personalized Exergaming Approach

HCPs expressed concerns about the technological challenges associated with exergaming, noting that some patients, particularly older adults, may lack the necessary skills or confidence to use exergaming technology effectively. This can be a barrier to exergaming’s successful implementation. HCPs emphasized the importance of familiarizing patients with exergaming technology to ensure its successful adoption. Furthermore, socialization (eg, involving family members in the exergaming process) was seen as an important facilitator of engagement. Opinions diverged with regard to the influence of patients’ sex on the ease of adopting exergaming. Some HCPs believed that men might find exergaming easier due to a perceived greater familiarity with video games or technology. Conversely, others suggested that women might have an advantage, particularly in using mobile phones and apps, which are often integral to mobile exergaming.

## Discussion

Per HCPs, while exergaming has the potential to motivate patients to become more physically active, it should be used in conjunction with other interventions (eg, cardiac rehabilitation). This study also highlighted potential challenges related to technology use. HCPs expressed concerns about the digital divide, particularly for older patients who may be less familiar with exergaming technology, as recently described in a systematic review on older adults’ experiences with exergaming [14]. There is a need for adequate familiarization and for tailoring exergaming experiences to individual patient characteristics.

## Supplementary material

10.2196/69126 Multimedia Appendix 1 Interview guide.

## Abbreviations:

GDPR: General Data Protection Regulation
HCP: health care professional
HF: heart failure
PA: physical activity
RCT: randomized controlled trial
