# Supplementary material for: Mobile Exergames to Reduce Sedentary Time in Patients With Heart Failure: What Do Health Care Professionals Expect?
Source: JMIR Serious Games. 2025 Nov 26;13:e69126. doi: 10.2196/69126 (PMC12661590; doi:10.2196/69126)
Supplement: Multimedia Appendix 1 [file games-v13-e69126-s001.docx]

**Interview guide**

As part of a semi-structured interview on the experience of healthcare professionals on sex differences in exercise in patients with heart failure, HCPs were asked one additional question to explore the expectations of HCPs regarding mobile exergaming as a tool to decrease sedentary time in patients with HF.

Introduction to this additional question

*Ehealth interventions, for example an app or mobile games, are tools that can be used to improve self-care behavior, such as physical activity in patients with heart failure. The Heart-eXg study is an example of such. The exergame is called “Heart farming” and uses AR motions to track the players motions.*

Question: Do you think an exergame might be suitable for patients with heart failure?

Follow-up questions such as what do you mean by that and could you give an example, were used in which the health care professionals were asked to develop their descriptions.
